# Interocular symmetry of retinal and choroidal OCT parameters in precapillary pulmonary hypertension: a brief research report

**DOI:** 10.3389/fmed.2026.1788867

**Published:** 2026-09-30

**Authors:** Diana A. Dmuchowska, Katarzyna Ptaszyńska, Joanna Konopińska, Karol Kamiński

**Affiliations:** 1 Department of Ophthalmology, Medical University of Bialystok, Bialystok, Poland; 2 Department of Cardiology and Internal Diseases, Medical University of Bialystok, Bialystok, Poland

**Keywords:** choroid, choroidal vascularity index, interocular symmetry, oculomics, optical coherence tomography, pulmonary hypertension, retina

## Abstract

**Purpose:**

To assess interocular symmetry of retinal and choroidal optical coherence tomography (OCT)-derived parameters in patients with precapillary pulmonary hypertension (PH) and healthy controls, and to evaluate the implications of symmetry for oculomics-based biomarker development.

**Methods:**

In this cross-sectional study, 29 patients with precapillary PH and 37 age- and sex-matched healthy controls underwent standardized spectral-domain OCT. Retinal and choroidal thicknesses, volumes and binarized choroidal biomarkers were quantified using validated protocols. Interocular agreement was assessed with intraclass correlation coefficients (ICC), Spearman's rank correlation (*ρ*), and paired testing with false discovery rate control.

**Results:**

Median interocular agreement was good but parameter-specific (ICC 0.76 in precapillary PH and 0.78 in controls). In PH, agreement was highest for choroidal metrics. In controls, it was lowest for retinal pigment epithelium thickness. No systematic laterality bias was detected in PH, whereas two parameters remained significant in controls. Absolute inter-eye differences were small but greater in PH for selected temporal and nasal retinal thickness and volume subfields.

**Conclusions:**

Interocular agreement was parameter-specific in both groups. In precapillary PH, one-eye sampling appears most defensible for choroidal biomarkers. Selected retinal parameters showed poor agreement, or greater absolute differences in precapillary PH than in controls, and require individual validation.

## 1 Introduction

Retinal imaging has emerged as a scalable and non-invasive gateway to systemic biomarker discovery, giving rise to the rapidly developing field of oculomics, which integrates quantitative ocular imaging with systemic phenotyping and predictive modelling. Review-level evidence shows that retinal and choroidal imaging features can encode information relevant to systemic cardiovascular and other non-ophthalmic conditions, supporting the concept of the eye as a window into whole-body health (1–3).

Pulmonary hypertension (PH) is a heterogeneous, progressive cardiopulmonary vasculopathy associated with pulmonary vascular remodelling, endothelial dysfunction, and right ventricular failure, with haemodynamic definitions and classification reflected in contemporary European Society of Cardiology (ESC)/European Respiratory Society (ERS) guidance (4). Importantly, PH is increasingly viewed as a systemic disorder with extrapulmonary end-organ involvement (5). Ocular manifestations are thought to arise primarily from altered ocular perfusion and venous congestion, resulting in choroidal changes and a spectrum of retinal complications (6).

The choroid—owing to its exceptionally high blood flow is a plausible downstream sensor of systemic haemodynamic and microvascular disturbance (7). Conceptual models of venous-overload choroidopathy provide a mechanistic framework for how sustained venous congestion can drive choroidal remodelling and serous retinal changes (6, 8). In parallel, quantitative optical coherence tomography (OCT)-derived biomarkers including choroidal thickness, volume and the choroidal vascularity index (CVI) have gained interest as interpretable markers of luminal–stromal remodelling and vascular congestion (9).

Prior imaging studies indicate that PH is associated with measurable retinal and chorioretinal microvascular alterations. OCT angiography (OCTA) data in primary PH demonstrated microvascular and neural changes, supporting a neurovascular signature beyond the pulmonary circulation (10). In chronic thromboembolic PH, OCT/OCTA studies reported retinal and choroidal perfusion abnormalities (11).

Importantly, the present cohort has already been characterized in terms of choroidal morphology and vascular structure. In a previous cross-sectional analysis performed in the same population of patients with precapillary PH, we demonstrated significantly reduced choroidal thickness, volume and choroidal vascularity index compared with healthy controls, together with limited correlations between ocular and invasive cardiological parameters. These findings supported the concept of disease-specific choroidal remodeling in PH and indicated that choroidal biomarkers may reflect systemic vascular dysfunction rather than isolated ocular changes. However, that analysis was performed at the group level and did not address whether these choroidal alterations are symmetrically expressed between fellow eyes, an assumption that underpins most oculomics-based biomarker approaches (12). In parallel, we also evaluated thicknesses of individual retinal layers and compared patients with precapillary PH with healthy controls, observing selective thinning of specific outer retinal layers in the disease group (13).

A core methodological assumption in many oculomics studies is interocular symmetry, which supports one-eye sampling strategies and informs handling of within-subject correlation when both eyes are analysed. Evidence from systemic vascular disease suggests that symmetry is often preserved, yet agreement can be parameter-specific and may vary with phenotype. In systemic sclerosis, interocular symmetry of macular and peripapillary choroidal parameters (including CVI and thickness) was confirmed, with the lowest correlations observed for CVI (14). Complementary evidence from diabetic retinopathy indicates that while choroidal thickness may be treated as representative from one eye, CVI symmetry can be reduced in the presence of diabetic macular oedema, suggesting that disease activity can selectively disrupt bilateral concordance of vascular biomarkers (15).

Against this background, the present study aimed to systematically quantify interocular symmetry of retinal and choroidal OCT parameters in patients with precapillary PH and healthy controls, to identify features with robust bilateral concordance suitable for systemic biomarker applications.

## 2 Materials and methods

### 2.1 Study design and participants

This was a prospective, single-center, cross-sectional study conducted at the Medical University of Bialystok. Twenty-nine consecutive adults with confirmed precapillary PH and 37 age- and sex-matched healthy controls were enrolled. Diagnosis of precapillary PH was established by right-heart catheterization in accordance with ESC criteria. As recruitment was initiated before publication of the 2022 ESC/ERS update, precapillary PH was defined according to criteria in force at the time as mean pulmonary arterial pressure (mPAP) ≥ 25 mmHg, pulmonary arterial wedge pressure (PAWP) ≤ 15 mmHg, and pulmonary vascular resistance (PVR) > 3 Wood units (16).

Inclusion criteria for both groups were age ≥ 18 years and written informed consent to participate in the study. Participants of the precapillary PH group fulfilled the ESC criteria (16). Controls were self-reported healthy individuals without a history of systemic or ocular disease. Exclusion criteria for both groups were ametropia ≥ 3 diopters; history of posterior segment surgery or intravitreal injections; cataract surgery performed less than 12 months before the examination; ocular or systemic disorders potentially affecting the choroid; prior retinal laser treatment; any fundus pathology or macular changes; and OCT scan quality < 25 dB.

Detailed clinical characterization, cardiological work-up, and the complete ophthalmic acquisition protocol have been reported previously (12) and are referenced here to avoid duplication.

The study adhered to the tenets of the Declaration of Helsinki and was approved by the Bioethics Committee of the Medical University of Bialystok (APK.002.290.2020). Written informed consent was obtained from all participants.

### 2.2 Ophthalmological examination

All patients underwent a comprehensive ophthalmological examination. The examination included refraction, best corrected visual acuity (BCVA) assessed using a Snellen chart and converted to logMAR, and slit-lamp biomicroscopy. Axial length and pachymetry were measured using a Tomey OA-2000 biometer (Nagoya, Japan). Intraocular pressure (IOP) was assessed with a Pascal dynamic contour tonometer (Ziemer Ophthalmic Systems AG, Port, Switzerland). Fundus evaluation was performed following pharmacological dilation. Spectral domain-OCT (Spectralis, Heidelberg Engineering GmbH, Heidelberg, Germany, 2016) was used to assess the retina and choroid. Blood pressure was measured after five minutes of seated rest.

### 2.3 Ophthalmic imaging

Spectral-domain OCT was performed using standardized macular protocols (Early Treatment Diabetic Retinopathy Study, ETDRS grid). Retinal thickness and volume metrics were extracted for total retina and predefined layers. The inner retina was defined as the region between the internal limiting membrane and the outer boundary of the outer plexiform layer; the outer nuclear layer (ONL) as the region between the outer boundary of the outer plexiform layer and the external limiting membrane; and the outer retinal layer (ORL) as the region extending from the external limiting membrane to Bruch's membrane, followed by the retinal pigment epithelium (RPE). Central retinal thickness refers to the point measurement at the foveal center, whereas central macular thickness refers to the mean retinal thickness within the central 500-µm radius of the ETDRS subfield. Throughout this manuscript, “outer retinal layer” (ORL) denotes this single slab as defined by the Spectralis (Heidelberg Engineering) segmentation, whereas “outer retinal layers” denotes the outer retinal compartment collectively, comprising the ONL, the ORL and the RPE. “Inner” and “Outer” in ETDRS subfield names (for example Inner T, Outer N) denote the topographic en-face rings of the ETDRS grid and not retinal depth. Choroidal metrics included choroidal thickness and volume in ETDRS subfields. Macular choroidal vascularity index (mCVI) was derived from standardized binarization of transfoveal Enhanced Depth Imaging (EDI)-OCT B-scans.

Retinal thickness and volume parameters were obtained automatically using the built-in Spectralis segmentation software. Choroidal thickness and binarized choroidal biomarkers were derived semi-automatically following previously published protocols. Choroidal parameters analyzed in the present study were retrieved from a previously established dataset generated in our earlier investigations of macular choroidal metrics in patients with precapillary PH and healthy controls, in which the full imaging and processing methodology has been described in detail (12).

The central macular region was defined as the central ETDRS subfield, corresponding to a circular area with a 500-µm radius centered on the fovea. All binarized choroidal measurements, including the mCVI, macular total choroidal area (mTCA), and macular luminal area (mLA), were performed independently by two evaluators blinded to clinical data using ImageJ software (NIH, USA) following the protocols of Sonoda et al. (17), and Agrawal et al. (18), with the step-by-step implementation described in detail in our previous publication (19). Inter-rater reproducibility was excellent, with intraclass correlation coefficients exceeding 0.90 for all binarized choroidal metrics. Given this high level of agreement, the mean of both evaluators’ measurements was used for subsequent analyses.

### 2.4 Cardiological evaluation

Cardiological metrics included clinical characteristics, right-heart catheterization, echocardiographic and laboratory parameters, as well as pharmacotherapy and the etiology of precapillary PH (Supplementary Table S1).

### 2.5 Statistical analysis

Data are presented as *n* (% of group) for nominal variables or as mean ± SD or median (Q1; Q3) for continuous variables, as appropriate. Normality of distribution was assessed using the Shapiro–Wilk test as well as skewness and kurtosis values. Group comparisons were performed using Fisher exact test or chi-square test for nominal data, Welch t-test or Mann–Whitney U test for continuous data, as appropriate. Benjamini–Hochberg (BH) correction for multiple comparisons was applied to control the false discovery rate at 5%, and both unadjusted and BH-adjusted *p*-values were reported. Benjamini–Hochberg adjustment was performed using the p.adjust function in R.

Interocular symmetry was evaluated across four complementary analytical dimensions: (1) interocular agreement using intraclass correlation coefficients (ICC); (2) monotonic inter-eye association using Spearman’s rank correlation coefficients (*ρ*); (3) systematic laterality bias using paired Wilcoxon signed-rank tests; and (4) degree of interocular asymmetry by comparing absolute inter-eye differences between patients with precapillary PH and healthy controls using the Mann–Whitney U test.

Interocular agreement was quantified using intraclass correlation coefficients (ICC) calculated with a two-way random-effects model, absolute-agreement definition, and single-measurement form. This model was selected to account for random effects of both subjects and eyes and to directly quantify absolute interocular concordance rather than consistency. Monotonic inter-eye associations were assessed with Spearman's rank correlation, and paired Wilcoxon signed-rank tests were used to test for systematic laterality bias. False discovery rate was controlled using the Benjamini–Hochberg procedure applied separately within predefined groups of related parameters (total retinal thickness metrics, retinal volume metrics, ORL thickness metrics, RPE thickness metrics, ONL thickness metrics, choroidal thickness metrics, choroidal volume metrics, and other choroidal parameters). ICC estimates were interpreted together with their 95% confidence intervals rather than as point values, and were categorised as poor (<0.50), moderate (0.50–0.75), good (0.75–0.90) or excellent (>0.90) (20–23). To assess whether systemic haemodynamic and vasoactive factors could influence the magnitude of interocular differences, additional sensitivity analyses using multivariable regression were performed. For each parameter, absolute percentage interocular asymmetry was calculated as |Left−Right|/[(Left + Right)/2] × 100. In the entire cohort, separate models included study group, mean arterial pressure (MAP) and caffeine consumption; a second analysis restricted to the PH group included MAP, caffeine consumption and sildenafil use. HC3 heteroskedasticity-robust standard errors were used and p-values were adjusted by the Benjamini–Hochberg procedure.

The statistical analysis was conducted using R version 4.2.1 (R Foundation for Statistical Computing, Vienna, Austria). All calculations were based on *α* = 0.05.

## 3 Results

### 3.1 Baseline characteristics

Baseline demographic and systemic characteristics are summarized in Table 1. The groups were overall comparable with respect to age and sex distribution. Patients with precapillary PH had lower diastolic blood pressure and mean arterial pressure compared with healthy controls and reported lower caffeine consumption. In contrast, baseline ocular parameters were highly comparable between right and left eyes within each group (Table 2), indicating preserved symmetry of basic refractive, biometric and keratometric characteristics. Detailed clinical, functional and echocardiographic characteristics of the precapillary PH cohort are summarized in Supplementary Table S1. The group was characterized by a median disease duration of 5 years, moderately elevated N-terminal pro–B-type natriuretic peptide (NT-proBNP) concentrations, preserved functional capacity in the majority of patients, and echocardiographic features consistent with precapillary PH, together with heterogeneous pharmacotherapy and etiological background.

**Table 1:** Baseline demographic and systemic characteristics of patients with precapillary pulmonary hypertension and healthy controls.

Variable	Total	Precapillary PH group	Control group	*p*
Number of patients	66	29	37	
Age (years)	52.62 ± 12.64	55.38 ± 15.40	50.46 ± 9.65	0.140
Sex, *n* (%)				
Female	34 (51.5)	13 (44.8)	21 (56.8)	0.475
Male	32 (48.5)	16 (55.2)	16 (43.2)	
BP systolic (mmHg)	122.58 ± 17.41	118.41 ± 11.87	126.48 ± 20.78	0.069
BP diastolic (mmHg)	77.53 ± 10.64	73.41 ± 9.24	81.39 ± 10.55	0.003
MAP (mmHg)	92.55 ± 11.31	88.41 ± 8.75	96.42 ± 12.17	**0** **.** **005**
Caffeine, *n* (%)	37 (56.1)	11 (37.9)	26 (70.3)	**0**.**017**
Nicotine (yes), *n* (%)	10 (15.2)	4 (13.8)	6 (16.2)	>0.999^a^

Data are presented as mean ± standard deviation for continuous variables and as number (percentage) for categorical variables. *P* values refer to comparisons between patients with precapillary PH and healthy controls. Groups compared with t-test for continuous variables and with chi-square test or Fisher's exact test^a^ for nominal variables.

BP, blood pressure; MAP, mean arterial pressure; PH, pulmonary hypertension.

*p* < 0.05 in bold.

**Table 2:** Baseline ocular characteristics stratified by eye in patients with precapillary pulmonary hypertension and healthy controls.

Parameter	Precapillary PH group – right eye	Precapillary PH group – left eye	*p* (Wilcoxon, BH)	Control group – right eye	Control group – left eye	*p* (Wilcoxon, BH)
AXL (mm)	23.48 ± 1.33	23.53 ± 1.08	0.971	23.32 ± 1.00	23.38 ± 0.96	0.921
logMAR	0.00 (0.00; 0.00)	0.00 (0.00; 0.00)	0.971	0.00 (0.00; 0.00)	0.00 (0.00; 0.00)	0.921
SE (D)	0.00 (−0.06; 1.00)	0.00 (−0.25; 0.50)	0.971	0.50 (−0.50; 1.75)	0.50 (−0.25; 1.50)	0.921
IOP (mmHg)	15.43 ± 3.50	15.65 ± 4.39	0.971	15.44 ± 2.36	15.29 ± 1.86	0.921
K1 (mm)	7.89 ± 0.24	7.87 ± 0.28	0.971	7.88 ± 0.29	7.88 ± 0.29	0.921
K2 (mm)	7.69 ± 0.27	7.69 ± 0.27	0.971	7.74 ± 0.30	7.74 ± 0.29	0.921

Data are presented as mean ± standard deviation for normally distributed variables (AXL, IOP, K1, K2) and as median (Q1; Q3) for non-normally distributed variables (logMAR, SE).

*P* values refer to paired comparisons between right and left eyes within each group using the Wilcoxon signed-rank test with Benjamini–Hochberg correction.

AXL, axial length; D, diopters; IOP, intraocular pressure; K1, K2, keratometry readings 1 and 2; logMAR, logarithm of the minimum angle of resolution; PH, pulmonary hypertension; SE, spherical equivalent.

### 3.2 Interocular symmetry of retinal and choroidal OCT parameters

Supplementary Tables S2, S3 provide descriptive inter-eye differences, whereas Tables 3, 4 summarize interocular agreement (ICC), monotonic inter-eye association (Spearman's *ρ*), and tests for systematic laterality bias (paired Wilcoxon). In the precapillary PH group, most retinal and choroidal parameters demonstrated at least moderate interocular agreement, with individual values ranging from poor to good-excellent, and moderate-to-strong monotonic inter-eye correlations. No parameter showed a statistically significant systematic laterality bias after Benjamini–Hochberg correction. Agreement was highest for choroidal metrics (median ICC 0.82, with 21 of 24 parameters reaching at least good agreement) and somewhat lower for retinal metrics (median 0.56). The lowest overall ICC was observed for total retinal thickness in the inferior outer ETDRS subfield (ICC = 0.130), notwithstanding a moderate-strong monotonic inter-eye association for the same parameter (*ρ* = 0.608, *p* < 0.001), suggesting preserved inter-eye ranking despite poor absolute agreement. Restricted between-subject variability may have contributed to the low ICC. In controls, interocular agreement was generally high, with the exception of RPE thickness, for which agreement was poor across all subfields. After Benjamini–Hochberg correction, a systematic laterality bias persisted for two ONL thickness parameters in the inner ring; no other parameter reached significance in either group.

**Table 3:** Interocular agreement, association, and laterality bias of retinal and choroidal OCT parameters in patients with precapillary pulmonary hypertension.

Variable	ICC between fellow eyes	95% CI for ICC	*ρ* between both eyes	*p*-value for *ρ*	*p*-value of Wilcoxon test (w/o correction)	*p*-value of Wilcoxon test (BH correction)
Total retinal thickness (μm)						
Outer T	0.353	0.009–0.635	0.415	**0** **.** **028**	0.178	0.444
Inner T	0.649	0.364–0.822	0.716	**<0**.**001**	0.031	0.310
Central macular	0.324	0.053–0.619	0.499	**0**.**007**	0.622	0.691
Outer N	0.405	0.046–0.672	0.448	**0**.**017**	0.445	0.556
Inner N	0.558	0.249–0.766	0.622	**<0**.**001**	0.311	0.444
Outer S	0.501	0.157–0.735	0.822	**<0**.**001**	0.274	0.444
Inner S	0.465	0.128–0.709	0.759	**<0**.**001**	0.222	0.444
Outer I	0.130	–0.256–0.477	0.608	**<0**.**001**	0.253	0.444
Inner I	0.826	0.657–0.915	0.853	**<0**.**001**	0.943	0.943
Central retinal thickness	0.233	–0.143–0.552	0.433	**0**.**021**	0.307	0.444
Total retinal volume (mm^3^)						
Outer T	0.317	–0.050–0.611	0.384	**0**.**043**	0.224	0.448
Inner T	0.583	0.283–0.781	0.782	**<0**.**001**	0.031	0.140
Central macular	0.650	0.369–0.822	0.693	**<0**.**001**	0.774	0.774
Outer N	0.556	0.190–0.776	0.644	**<0**.**001**	0.016	0.140
Inner N	0.554	0.241–0.764	0.588	**0**.**001**	0.042	0.140
Outer S	0.818	0.648–0.911	0.843	**<0**.**001**	0.351	0.501
Inner S	0.856	0.715–0.931	0.894	**<0**.**001**	0.637	0.774
Outer I	0.196	–0.165–0.518	0.656	**<0**.**001**	0.113	0.283
Inner I	0.816	0.639–0.910	0.871	**<0**.**001**	0.716	0.774
Total	0.813	0.638–0.909	0.819	**<0**.**001**	0.339	0.501
ORL thickness (μm)						
Inner T	0.784	0.585–0.894	0.776	**<0**.**001**	0.907	0.907
Central macular	0.538	0.221–0.754	0.553	**0**.**002**	0.278	0.695
Inner N	0.675	0.409–0.835	0.648	**<0**.**001**	0.623	0.907
Inner S	0.756	0.510–0.883	0.707	**<0**.**001**	0.023	0.115
Inner I	0.662	0.386–0.829	0.616	**<0**.**001**	0.883	0.907
RPE thickness (μm)						
Inner T	0.679	0.418–0.837	0.698	**<0**.**001**	0.465	0.785
Central macular	0.471	0.130–0.714	0.542	**0**.**003**	0.312	0.785
Inner N	0.768	0.557–0.886	0.740	**<0**.**001**	0.785	0.785
Inner S	0.678	0.412–0.837	0.483	**0**.**009**	0.769	0.785
Inner I	0.759	0.545–0.881	0.778	**<0**.**001**	0.578	0.785
ONL thickness (μm)						
Inner T	0.547	0.220–0.763	0.593	**0**.**001**	0.791	0.864
Central macular	0.450	0.100–0.701	0.448	**0**.**017**	0.336	0.675
Inner N	0.501	0.160–0.734	0.505	**0**.**006**	0.864	0.864
Inner S	0.331	–0.043–0.623	0.432	**0**.**022**	0.237	0.675
Inner I	0.418	0.052–0.683	0.527	**0**.**004**	0.405	0.675
Choroidal thickness (μm)						
Outer T	0.774	0.572–0.888	0.774	**<0**.**001**	0.331	0.878
Inner T	0.832	0.670–0.918	0.814	**<0**.**001**	0.554	0.909
Central macular	0.812	0.636–0.908	0.802	**<0**.**001**	0.351	0.878
Outer N	0.862	0.725–0.934	0.874	**<0**.**001**	0.250	0.878
Inner N	0.772	0.563–0.888	0.777	**<0**.**001**	0.829	0.909
Outer S	0.874	0.747–0.940	0.840	**<0**.**001**	0.682	0.909
Inner S	0.785	0.586–0.895	0.768	**<0**.**001**	0.909	0.909
Outer I	0.768	0.551–0.887	0.771	**<0**.**001**	0.620	0.909
Inner I	0.899	0.795–0.952	0.898	**<0**.**001**	0.764	0.909
SFCT (μm)	0.784	0.588–0.894	0.754	**<0**.**001**	0.345	0.878
Choroidal volume (mm^3^)						
Outer T	0.765	0.556–0.883	0.770	**<0**.**001**	0.330	0.722
Inner T	0.817	0.645–0.911	0.804	**<0**.**001**	0.737	0.879
Central macular	0.833	0.673–0.919	0.816	**<0**.**001**	0.196	0.653
Outer N	0.732	0.503–0.866	0.869	**<0**.**001**	0.175	0.653
Inner N	0.492	0.147–0.729	0.791	**<0**.**001**	0.361	0.722
Outer S	0.765	0.553–0.884	0.799	**<0**.**001**	0.811	0.879
Inner S	0.826	0.658–0.916	0.807	**<0**.**001**	0.879	0.879
Outer I	0.810	0.626–0.908	0.813	**<0**.**001**	0.551	0.879
Inner I	0.899	0.795–0.952	0.892	**<0**.**001**	0.841	0.879
Total	0.651	0.378–0.821	0.712	**<0**.**001**	0.168	0.653
Other choroidal parameters						
mTCA (μm^2^)	0.893	0.766–0.953	0.832	**<0**.**001**	0.731	0.754
mLA (μm^2^)	0.900	0.781–0.956	0.831	**<0**.**001**	0.360	0.720
mSA (μm^2^)	0.866	0.709–0.941	0.767	**<0**.**001**	0.754	0.754
mCVI (%)	0.828	0.634–0.923	0.787	**<0**.**001**	0.190	0.720

Data are presented as intraclass correlation coefficients (ICC) with 95% confidence intervals (95% CI), Spearman's rank correlation coefficients (*ρ*), and *p* values. Inter-eye differences were additionally evaluated using the paired Wilcoxon signed-rank test to assess systematic laterality bias. *P* values were adjusted for multiple comparisons using the Benjamini–Hochberg procedure. BH correction was applied for each group of parameters separately (i.e., total retinal thickness parameters, total retinal volume parameters, ORL thickness parameters, RPE thickness parameters, ONL thickness parameters, choroidal thickness parameters, choroidal volume parameters, other choroidal parameters).

ETDRS subfields refer to the conventional Early Treatment Diabetic Retinopathy Study grid centred on the fovea: Central Macular – central subfield (within a 500-μm radius); Inner – inner ring (500–1500 μm); Outer – outer ring (1500–3000 μm). Thus “Inner T” denotes the temporal subfield of the inner ring. “Inner” and “Outer” denote topographic ETDRS rings and not retinal depth; retinal layers are designated separately as ONL, ORL and RPE.

CVI, choroidal vascularity index; I, inferior; mCVI, macular choroidal vascularity index; mLA, macular luminal area; mSA, macular stromal area; mTCA, macular total choroidal area; N, nasal; ONL, outer nuclear layer; ORL, outer retinal layer; PH, pulmonary hypertension; RPE, retinal pigment epithelium; SFCT, subfoveal choroidal thickness; S, superior; T, temporal.

*p* < 0.05 in bold.

**Table 4:** Interocular agreement, association, and laterality bias of retinal and choroidal OCT parameters in healthy controls.

Variable	ICC between both eyes	95% CI for ICC	*ρ* between both eyes	*p*-value for *ρ*	*p*-value of Wilcoxon test (w/o correction)	*p*-value of Wilcoxon test (BH correction)
Total retinal thickness (μm)						
Outer T	0.835	0.692–0.913	0.893	**<0** **.** **001**	0.028	0.140
Inner T	0.926	0.861–0.961	0.927	**<0**.**001**	0.930	0.999
Central macular	0.782	0.618–0.881	0.788	**<0**.**001**	0.809	0.999
Outer N	0.799	0.644–0.891	0.822	**<0**.**001**	0.353	0.588
Inner N	0.819	0.674–0.903	0.859	**<0**.**001**	0.237	0.554
Outer S	0.836	0.705–0.912	0.835	**<0**.**001**	0.265	0.554
Inner S	0.898	0.811–0.946	0.907	**<0**.**001**	0.999	0.999
Outer I	0.773	0.585–0.879	0.845	**<0**.**001**	0.028	0.140
Inner I	0.795	0.636–0.889	0.872	**<0**.**001**	0.277	0.554
Central retinal thickness	0.560	0.292–0.747	0.684	**<0**.**001**	0.632	0.903
Total retinal volume (mm^3^)						
Outer T	0.845	0.709–0.919	0.893	**<0**.**001**	0.022	0.220
Inner T	0.918	0.847–0.957	0.937	**<0**.**001**	0.379	0.619
Central macular	0.727	0.530–0.850	0.769	**<0**.**001**	0.873	0.881
Outer N	0.933	0.875–0.965	0.933	**<0**.**001**	0.346	0.619
Inner N	0.898	0.812–0.946	0.859	**<0**.**001**	0.344	0.619
Outer S	0.835	0.703–0.912	0.833	**<0**.**001**	0.433	0.619
Inner S	0.871	0.764–0.932	0.893	**<0**.**001**	0.881	0.881
Outer I	0.778	0.603–0.881	0.844	**<0**.**001**	0.100	0.500
Inner I	0.804	0.651–0.894	0.888	**<0**.**001**	0.640	0.800
Total	0.527	0.247–0.725	0.879	**<0**.**001**	0.313	0.619
ORL thickness (μm)						
Inner T	0.562	0.293–0.748	0.559	**<0**.**001**	0.781	0.976
Central macular	0.527	0.248–0.725	0.618	**<0**.**001**	0.220	0.453
Inner N	0.742	0.554–0.858	0.783	**<0**.**001**	0.110	0.453
Inner S	0.651	0.416–0.804	0.647	**<0**.**001**	0.983	0.983
Inner I	0.781	0.617–0.881	0.805	**<0**.**001**	0.272	0.453
RPE thickness (μm)						
Inner T	0.353	0.038–0.605	0.338	**0**.**041**	0.379	0.533
Central macular	0.266	–0.050–0.537	0.218	0.196	0.208	0.520
Inner N	0.318	0.018–0.572	0.339	**0**.**040**	0.043	0.215
Inner S	0.448	0.144–0.673	0.407	**0**.**012**	0.875	0.875
Inner I	0.471	0.179–0.687	0.531	**0**.**001**	0.426	0.533
ONL thickness (μm)						
Inner T	0.694	0.457–0.834	0.807	**<0**.**001**	<0.001	**<0**.**001**
Central macular	0.812	0.663–0.899	0.866	**<0**.**001**	0.566	0.708
Inner N	0.610	0.338–0.783	0.643	**<0**.**001**	0.002	**0**.**005**
Inner S	0.636	0.395–0.794	0.560	**<0**.**001**	0.876	0.876
Inner I	0.804	0.651–0.894	0.804	**<0**.**001**	0.214	0.357
Choroidal thickness (μm)						
Outer T	0.774	0.583–0.880	0.770	**<0**.**001**	0.038	0.380
Inner T	0.797	0.640–0.890	0.745	**<0**.**001**	0.769	0.769
Central macular	0.777	0.610–0.878	0.779	**<0**.**001**	0.319	0.745
Outer N	0.708	0.504–0.838	0.656	**<0**.**001**	0.239	0.745
Inner N	0.818	0.673–0.903	0.765	**<0**.**001**	0.447	0.745
Outer S	0.787	0.620–0.885	0.792	**<0**.**001**	0.120	0.600
Inner S	0.788	0.626–0.884	0.766	**<0**.**001**	0.684	0.769
Outer I	0.782	0.615–0.882	0.748	**<0**.**001**	0.695	0.769
Inner I	0.729	0.534–0.850	0.661	**<0**.**001**	0.582	0.769
SFCT	0.661	0.431–0.810	0.707	**<0**.**001**	0.415	0.745
Choroidal volume (mm^3^)						
Outer T	0.747	0.532–0.867	0.766	**<0**.**001**	0.024	0.240
Inner T	0.791	0.630–0.887	0.739	**<0**.**001**	0.749	0.749
Central macular	0.770	0.598–0.874	0.781	**<0**.**001**	0.351	0.585
Outer N	0.821	0.681–0.903	0.784	**<0**.**001**	0.118	0.393
Inner N	0.805	0.655–0.894	0.765	**<0**.**001**	0.210	0.420
Outer S	0.788	0.617–0.886	0.807	**<0**.**001**	0.079	0.393
Inner S	0.787	0.625–0.884	0.763	**<0**.**001**	0.660	0.749
Outer I	0.759	0.580–0.868	0.724	**<0**.**001**	0.194	0.420
Inner I	0.752	0.569–0.864	0.694	**<0**.**001**	0.675	0.749
Total	0.872	0.767–0.932	0.833	**<0**.**001**	0.411	0.587
Other choroidal parameters						
mTCA (μm^2^)	0.794	0.635–0.889	0.787	**<0**.**001**	0.964	0.964
mLA (μm^2^)	0.782	0.616–0.882	0.723	**<0**.**001**	0.835	0.964
mSA (μm^2^)	0.788	0.625–0.885	0.814	**<0**.**001**	0.846	0.964
mCVI (%)	0.564	0.303–0.747	0.568	**<0**.**001**	0.208	0.832

Data are presented as intraclass correlation coefficients (ICC) with 95% confidence intervals (95% CI), Spearman's rank correlation coefficients (*ρ*), and *p* values. Inter-eye differences were additionally evaluated using the paired Wilcoxon signed-rank test to assess systematic laterality bias. *P* values were adjusted for multiple comparisons using the Benjamini–Hochberg procedure. BH correction was applied for each group of parameters separately (i.e., total retinal thickness parameters, total retinal volume parameters, ORL thickness parameters, RPE thickness parameters, ONL thickness parameters, choroidal thickness parameters, choroidal volume parameters, other choroidal parameters).

ETDRS subfields refer to the conventional Early Treatment Diabetic Retinopathy Study grid centred on the fovea: Central Macular – central subfield (within a 500-μm radius); Inner – inner ring (500–1500 μm); Outer – outer ring (1500–3000 μm). Thus “Inner T” denotes the temporal subfield of the inner ring. “Inner” and “Outer” denote topographic ETDRS rings and not retinal depth; retinal layers are designated separately as ONL, ORL and RPE.

CVI, choroidal vascularity index; I, inferior; mCVI, macular choroidal vascularity index; mLA, macular luminal area; mSA, macular stromal area; mTCA, macular total choroidal area; N, nasal; ONL, outer nuclear layer; ORL, outer retinal layer; PH, pulmonary hypertension; RPE, retinal pigment epithelium; SFCT, subfoveal choroidal thickness; S, superior; T, temporal.

*p* < 0.05 in bold.

In addition to agreement, inter-eye association, and laterality bias analyses, we evaluated the degree of interocular asymmetry by comparing absolute inter-eye differences of patients with precapillary PH versus healthy controls (Supplementary Table S4). Absolute inter-eye differences were small in both groups. After Benjamini–Hochberg correction, absolute inter-eye differences were greater in the precapillary PH group for total retinal thickness in the inner temporal and inner and outer nasal subfields, and for total retinal volume in the inner and outer temporal and nasal ETDRS subfields (Supplementary Table S4). No ONL, ORL or RPE thickness parameter and no choroidal parameter differed between groups. In sensitivity analyses, none of mean arterial pressure, caffeine consumption, or sildenafil use was consistently associated with the magnitude of interocular differences. Isolated associations were detected for single parameters, one in the whole cohort and one within the precapillary PH group, but neither was reproduced across related metrics. Complete results are given in Supplementary Tables S5, S6.

## 4 Discussion

This brief research report provides the first systematic assessment of interocular symmetry of retinal and choroidal OCT-derived biomarkers in patients with precapillary PH and healthy controls. It directly addresses a key methodological assumption underlying oculomics-based biomarker development, namely, whether OCT-derived ocular features can be treated as bilaterally redundant or require eye-specific consideration. Principal findings were threefold: (a) most retinal and choroidal variables demonstrated moderate-to-high interocular agreement in both groups; (b) no consistent laterality bias was detected after multiplicity correction, apart from two ONL parameters in controls; and (c) agreement was feature-dependent, indicating that symmetry should be treated as a parameter-specific property rather than a universal assumption.

Beyond interocular agreement metrics, the principal observation is that bilateral symmetry is maintained in the study cohort. Median interocular agreement was moderate-to-good but varied between individual parameters. The absolute differences between fellow eyes were small, and no systematic laterality bias was detected in the precapillary PH group, with only two ONL parameters reaching significance in controls. For the purposes of oculomics, whereas selected retinal parameters require parameter-level validation, one-eye sampling appears most defensible for choroidal metrics. This is the assumption most biomarker pipelines rely on and which had not previously been tested in PH. A secondary observation is that the magnitude of these small absolute inter-eye differences was somewhat greater in precapillary PH than in controls, and only for total retinal thickness and volume in the temporal and nasal subfields. No ONL, ORL, or RPE thickness parameter and no choroidal parameter differed. This is a difference in magnitude rather than clinically apparent asymmetry, and it should be interpreted cautiously. It may reflect the greater perfusion reserve of the choroid relative to the retinal circulation (7). In addition, the retinal vasculature is end-arterial and lacks collateral supply, so a systemic perfusion deficit may act focally and at locations that need not correspond between fellow eyes. The terminal retinal vessels are in this sense the compartment most exposed to ischaemic insult. We offer this only as a possibility. In our parallel structural analysis of the same cohort we observed selective thinning of the outer retinal layers and the choroid compared with healthy controls (12, 13). The present analysis extends those findings by quantifying the degree to which these biomarkers are symmetrically expressed between fellow eyes—an aspect that directly impacts study design and statistical modeling in oculomics.

Although most OCT-derived parameters showed good agreement between fellow eyes, selected retinal thickness and volume measures exhibited lower inter-eye concordance in patients with precapillary PH. Choroidal parameters, by contrast, were the most concordant of all compartments examined. Similar parameter-specific reductions in interocular symmetry have been reported in other systemic vasculopathies (14, 15). It cannot be determined from the present data whether the greater magnitude of absolute inter-eye differences observed here also constitutes an additional disease-related imaging marker.

Several limitations should be acknowledged. The modest sample sizes (*n* = 29 and *n* = 37) limit the precision of the ICC estimates. As the ICC depends on between-subject variability, restricted variance may yield low values even when absolute inter-eye differences are small. Low ICC values should therefore be interpreted together with their 95% confidence intervals and with the absolute inter-eye differences (Supplementary Tables S2–S4), and not as evidence of biological asymmetry. The precapillary PH group was etiologically heterogeneous. Subgroup analyses were not feasible given the small numbers within individual categories. The etiologies differ in the degree of chronic hypoxaemia and venous congestion imposed on the ocular circulations and in whether shunt physiology or chronic anticoagulation is present. Because these factors act systemically, they would not necessarily be expected to generate systematic laterality. Nevertheless, etiology-specific effects on the magnitude of inter-eye differences cannot be excluded, and larger studies are needed to determine whether interocular symmetry differs by PH etiology. Patients with precapillary PH had lower mean arterial pressure and caffeine consumption than controls, and a majority were receiving vasoactive medication. The sensitivity analyses showed no consistent influence of these factors. The absence of an association with sildenafil does not exclude a symmetric bilateral effect on absolute measurements. Finally, the cross-sectional design precludes causal inference, and replication in larger, independent cohorts will be required.

Strengths of the present study include standardized acquisition and processing workflows, inclusion of an age- and sex-matched control group, and a comprehensive, multi-dimensional framework for interocular symmetry assessment incorporating agreement, inter-eye association, laterality bias, and degree of asymmetry with false discovery rate control.

Collectively, our findings support one-eye sampling for choroidal measures in this disease, with choroidal metrics the most robust, and indicate that symmetry should be treated as a parameter-specific property when selecting features for oculomics studies. Longitudinal studies integrating OCT/OCTA features with invasive hemodynamics and clinical outcomes are warranted to define prognostic and monitoring utility.

## 5 Conclusions

In this cohort, interocular agreement of OCT-derived retinal and choroidal parameters was feature-specific in both precapillary PH and healthy controls. Choroidal biomarkers showed the most consistent bilateral agreement in precapillary PH, supporting one-eye sampling most of these measures. Selected retinal thickness and volume subfields showed poorer agreement or greater absolute inter-eye differences in precapillary PH than in controls, whereas no between-group differences in asymmetry were found for outer retinal layers or choroidal parameters. Interocular symmetry should therefore not be assumed universally but validated for each candidate oculomics biomarker. Confirmation in larger cohorts is required.

## Data Availability

The raw data supporting the conclusions of this article will be made available by the authors, without undue reservation.
