# Supplementary material for: Interocular symmetry of retinal and choroidal OCT parameters in precapillary pulmonary hypertension: a brief research report
Source: Front Med (Lausanne). 2026 Sep 30;13:1788867. doi: 10.3389/fmed.2026.1788867 (PMC13661351; doi:10.3389/fmed.2026.1788867)
Supplement: Supplementary file 1 [file Supplementaryfile1.docx]

**Table S1. Precapillary pulmonary hypertension-specific clinical, functional and echocardiographic characteristics, pharmacotherapy and etiology.**

| **Parameter** | **Value** |
| --- | --- |
| **Disease characteristics** | |
| Precapillary PH duration (years) | 5.00 (3.00;13.00) |
| NT-proBNP (pg/mL) | 227.55 (155.93;992.33) |
| Peripheral edema, n (%) | 4 (13.8) |
| 6MWT (m) | 437.38 ± 146.82 |
| **Echocardiography** | |
| TAPSE (mm) | 17.32 ± 5.68 |
| TR peak gradient (mmHg) | 53.22 ± 25.57 |
| eMPAP (mmHg) | 39.04 ± 16.00 |
| RAP (mmHg) | 5.90 ± 4.17 |
| RAA (cm²) | 20.00 (17.00;27.00) |
| RV diameter 4CH (cm) | 4.60 (3.85;5.00) |
| sPAP (mmHg) | 52.36 ± 26.71 |
| **Pharmacotherapy, n (%)** | |
| Sildenafil | 16 (55.2) |
| Bosentan | 11 (37.9) |
| Furosemide | 11 (37.9) |
| **Precapillary PH etiology, n (%)** | |
| Idiopathic | 16 (55.2) |
| Congenital | 8 (27.6) |
| CTEPH | 3 (10.3) |
| Surgical defect | 1 (3.4) |
| Non-surgical defect | 2 (6.9) |
| Connective tissue disease–associated | 1 (3.4) |

Data are presented as mean ± standard deviation for normally distributed variables, as median (Q1; Q3) for non-normally distributed variables, and as number (percentage) for categorical variables.

Abbreviations: 6MWT – 6-minute walk test; CTEPH – chronic thromboembolic pulmonary hypertension; eMPAP – estimated mean pulmonary artery pressure; NT-proBNP – N-terminal pro–B-type natriuretic peptide; PH – pulmonary hypertension; Q1 – first quartile; Q3 – third quartile; RAP – right atrial pressure; RAA – right atrial area; RV – right ventricle; 4CH – four-chamber view; sPAP – systolic pulmonary artery pressure; TAPSE – tricuspid annular plane systolic excursion; TR – tricuspid regurgitation.

**Table S2. Inter-eye descriptive differences of retinal and choroidal OCT parameters in patients with precapillary pulmonary hypertension.**

| **Variable** | **Left eye (mean ± SD)** | **Right eye (mean ± SD)** | **Median Left (Q1; Q3)** | **Median Right (Q1; Q3)** | **Relative mean difference (%)** |
| --- | --- | --- | --- | --- | --- |
| **Total retinal thickness (μm)** | | | | | |
| **Outer T** | 277.72±12.45 | 282.64±23.14 | 276.00 (270.00;286.00) | 282.00 (269.75;301.25) | -1.756 |
| **Inner T** | 311.41±17.84 | 318.07±22.62 | 314.00 (300.00;325.00) | 321.00 (309.00;338.25) | -2.115 |
| **Central Macular** | 261.86±22.26 | 264.71±21.04 | 262.00 (250.00;274.00) | 264.00 (256.00;279.00) | -1.083 |
| **Outer N** | 297.97±24.60 | 293.82±21.85 | 302.00 (286.00;314.00) | 293.50 (273.75;316.00) | 1.401 |
| **Inner N** | 326.38±16.21 | 320.68±25.96 | 328.00 (314.00;341.00) | 325.00 (302.75;342.25) | 1.762 |
| **Outer S** | 287.14±22.41 | 288.04±18.03 | 291.00 (278.00;298.00) | 292.00 (273.50;302.25) | -0.312 |
| **Inner S** | 327.93±16.52 | 322.21±27.14 | 329.00 (315.00;341.00) | 326.50 (308.25;339.25) | 1.759 |
| **Outer I** | 282.10±29.69 | 277.89±17.51 | 282.00 (271.00;293.00) | 281.00 (264.75;289.25) | 1.504 |
| **Inner I** | 324.07±16.06 | 324.00±19.68 | 328.00 (312.00;338.00) | 327.00 (309.50;338.00) | 0.021 |
| **Central Retinal Thickness** | 233.69±27.38 | 228.18±24.72 | 230.00 (214.00;241.00) | 225.50 (208.25;245.25) | 2.386 |
| **Total retinal volume (mm³)** | | | | | |
| **Outer T** | 1.47±0.07 | 1.50±0.14 | 1.46 (1.43;1.51) | 1.50 (1.43;1.60) | -1.948 |
| **Inner T** | 0.49±0.03 | 0.50±0.05 | 0.49 (0.47;0.51) | 0.51 (0.49;0.53) | -1.874 |
| **Central Macular** | 0.21±0.02 | 0.21±0.02 | 0.21 (0.20;0.22) | 0.21 (0.20;0.22) | 0.539 |
| **Outer N** | 1.59±0.08 | 1.54±0.10 | 1.59 (1.55;1.64) | 1.51 (1.45;1.62) | 3.102 |
| **Inner N** | 0.52±0.03 | 0.51±0.03 | 0.52 (0.49;0.53) | 0.51 (0.50;0.53) | 2.191 |
| **Outer S** | 1.54±0.07 | 1.53±0.10 | 1.54 (1.48;1.58) | 1.55 (1.45;1.60) | 0.726 |
| **Inner S** | 0.51±0.03 | 0.51±0.03 | 0.52 (0.50;0.54) | 0.52 (0.50;0.53) | 0.389 |
| **Outer I** | 1.51±0.12 | 1.47±0.09 | 1.49 (1.44;1.55) | 1.49 (1.40;1.53) | 2.563 |
| **Inner I** | 0.51±0.02 | 0.51±0.03 | 0.52 (0.49;0.53) | 0.52 (0.49;0.53) | -0.131 |
| **Total** | 8.34±0.35 | 8.28±0.47 | 8.40 (8.17;8.59) | 8.39 (7.94;8.59) | 0.795 |
| **ORL thickness (μm)** | | | | | |
| **Inner T** | 79.07±2.51 | 79.00±2.78 | 79.00 (78.00;80.00) | 79.00 (77.00;81.00) | 0.087 |
| **Central Macular** | 84.76±3.29 | 83.86±3.63 | 84.00 (82.00;87.00) | 85.00 (81.75;86.00) | 1.069 |
| **Inner N** | 80.00±2.48 | 79.86±2.86 | 79.00 (78.00;82.00) | 80.00 (78.00;81.25) | 0.179 |
| **Inner S** | 79.45±2.15 | 78.71±2.73 | 79.00 (78.00;81.00) | 78.50 (76.00;80.00) | 0.928 |
| **Inner I** | 78.28±2.75 | 78.32±3.23 | 78.00 (76.00;80.00) | 78.00 (75.75;80.25) | -0.058 |
| **RPE thickness (μm)** | | | | | |
| **Inner T** | 13.62±1.32 | 13.79±1.37 | 14.00 (13.00;14.00) | 14.00 (13.00;15.00) | -1.204 |
| **Central Macular** | 15.38±1.35 | 15.11±1.26 | 15.00 (15.00;16.00) | 15.00 (14.00;16.00) | 1.785 |
| **Inner N** | 14.21±1.70 | 14.14±1.88 | 14.00 (13.00;16.00) | 14.00 (13.00;15.00) | 0.452 |
| **Inner S** | 14.17±1.47 | 14.07±1.74 | 14.00 (13.00;15.00) | 14.00 (13.00;15.00) | 0.715 |
| **Inner I** | 13.55±1.53 | 13.68±1.81 | 13.00 (13.00;14.00) | 13.50 (12.00;14.25) | -0.932 |
| **ONL thickness (μm)** | | | | | |
| **Inner T** | 69.24±6.27 | 69.21±9.25 | 69.00 (64.00;74.00) | 71.00 (63.00;76.00) | 0.039 |
| **Central Macular** | 86.90±9.42 | 88.79±10.74 | 88.00 (83.00;91.00) | 89.50 (83.00;96.25) | -2.151 |
| **Inner N** | 68.17±12.68 | 69.32±8.79 | 70.00 (64.00;77.00) | 72.50 (61.00;76.00) | -1.671 |
| **Inner S** | 62.24±10.26 | 64.61±11.36 | 62.00 (55.00;68.00) | 67.50 (58.75;73.25) | -3.730 |
| **Inner I** | 65.31±9.70 | 64.68±7.63 | 67.00 (62.00;72.00) | 65.00 (59.75;70.00) | 0.972 |
| **Choroidal thickness (μm)** | | | | | |
| **Outer T** | 215.21±71.28 | 223.82±82.64 | 223.00 (157.00;285.00) | 236.00 (146.75;285.75) | -3.924 |
| **Inner T** | 239.41±94.85 | 242.04±96.22 | 235.00 (170.00;302.00) | 246.00 (155.00;312.00) | -1.089 |
| **Central Macular** | 245.72±96.15 | 249.54±106.78 | 239.00 (166.00;306.00) | 246.50 (155.50;334.25) | -1.539 |
| **Outer N** | 194.76±77.82 | 202.89±82.96 | 193.00 (123.00;258.00) | 200.00 (131.25;254.50) | -4.091 |
| **Inner N** | 230.76±93.30 | 227.11±99.45 | 222.00 (154.00;314.00) | 209.50 (142.75;316.25) | 1.595 |
| **Outer S** | 254.34±97.65 | 254.43±96.04 | 264.00 (165.00;315.00) | 257.00 (162.50;330.00) | -0.033 |
| **Inner S** | 246.03±103.31 | 247.32±102.05 | 233.00 (167.00;338.00) | 233.50 (159.25;321.25) | -0.522 |
| **Outer I** | 228.43±86.80 | 223.61±84.61 | 241.50 (159.50;298.00) | 224.00 (150.00;292.00) | 2.133 |
| **Inner I** | 234.00±102.23 | 234.36±96.89 | 239.00 (159.00;327.00) | 230.00 (156.50;314.00) | -0.153 |
| **SFCT** | 240.86±102.29 | 247.96±108.37 | 234.00 (147.00;317.00) | 250.50 (153.25;331.50) | -2.906 |
| **Choroidal volume (mm³)** | | | | | |
| **Outer T** | 1.14±0.38 | 1.18±0.44 | 1.18 (0.83;1.51) | 1.26 (0.78;1.51) | -3.479 |
| **Inner T** | 0.38±0.15 | 0.38±0.16 | 0.37 (0.27;0.48) | 0.37 (0.25;0.50) | -1.289 |
| **Central Macular** | 0.19±0.08 | 0.20±0.09 | 0.19 (0.12;0.24) | 0.20 (0.12;0.26) | -3.873 |
| **Outer N** | 1.02±0.42 | 1.09±0.46 | 0.95 (0.65;1.37) | 1.04 (0.73;1.34) | -6.793 |
| **Inner N** | 0.35±0.15 | 0.33±0.24 | 0.35 (0.21;0.49) | 0.34 (0.23;0.50) | 6.187 |
| **Outer S** | 1.30±0.49 | 1.31±0.50 | 1.34 (0.87;1.67) | 1.31 (0.86;1.59) | -1.090 |
| **Inner S** | 0.39±0.16 | 0.38±0.17 | 0.37 (0.26;0.54) | 0.37 (0.23;0.50) | 1.228 |
| **Outer I** | 1.19±0.46 | 1.19±0.45 | 1.22 (0.85;1.58) | 1.19 (0.79;1.55) | 0.600 |
| **Inner I** | 0.37±0.16 | 0.37±0.15 | 0.37 (0.25;0.52) | 0.37 (0.24;0.49) | 0.117 |
| **Total** | 6.32±2.45 | 6.85±2.50 | 6.15 (3.91;8.77) | 7.14 (4.52;8.39) | -8.013 |
| **Other choroidal parameters** | | | | | |
| **mTCA (μm²)** | 309 403.69±99 364.52 | 329 835.60±118 839.49 | 328852.00 (255 337.75;353 114.00) | 354778.50 (274 174.88;400 403.13) | -6.393 |
| **mLA (μm²)** | 202 084.48±65 178.00 | 216 557.50±77 753.14 | 211258.50 (168 111.25;236 095.25) | 226187.25 (180 921.38;266 288.50) | -6.914 |
| **mSA (μm²)** | 107 319.20±35 725.86 | 113 278.10±42 149.16 | 111428.50 (86 553.25;128 460.50) | 122010.50 (91 668.75;134 308.38) | -5.403 |
| **mCVI (%)** | 65.33±2.86 | 65.68±2.87 | 65.23 (63.11;67.46) | 66.07 (64.11;67.17) | -0.539 |

Relative mean difference (%) was calculated as the percent difference between left and right eyes normalized to their mean:

$$\text{Relative mean difference (\%) }=\frac{\text{Left - Right}}{(\text{Left + Right})/2}\times100$$

ETDRS subfields refer to the conventional Early Treatment Diabetic Retinopathy Study grid centered on the fovea: Central Macular – central subfield (within a 500-μm radius); Inner – inner ring (500–1500 μm); Outer – outer ring (1500–3000 μm). Thus “Inner T” denotes the temporal subfield of the inner ring. “Inner” and “Outer” denote topographic ETDRS rings and not retinal depth; retinal layers are designated separately as ONL, ORL and RPE.

Abbreviations: CVI – choroidal vascularity index; I – inferior; mCVI – macular choroidal vascularity index; mLA – macular luminal area; mSA – macular stromal area; mTCA – macular total choroidal area; N – nasal; OCT – optical coherence tomography; ONL – outer nuclear layer; ORL – outer retinal layer; Q1 – first quartile; Q3 – third quartile; RPE – retinal pigment epithelium; SD – standard deviation; SFCT – subfoveal choroidal thickness; S – superior; T – temporal.

**Table S3. Inter-eye descriptive differences of retinal and choroidal OCT parameters in healthy controls.**

| **Variable** | **Left eye (mean ± SD)** | **Right eye (mean ± SD)** | **Median Left (Q1; Q3)** | **Median Right (Q1; Q3)** | **Relative mean difference (%)** |
| --- | --- | --- | --- | --- | --- |
| **Total retinal thickness (μm)** | | | | | |
| **Outer T** | 281.30±11.12 | 283.59±11.93 | 280.00 (274.00;286.00) | 283.00 (274.00;293.00) | -0.813 |
| **Inner T** | 326.97±11.29 | 327.41±11.19 | 327.00 (319.00;335.00) | 326.00 (320.00;336.00) | -0.132 |
| **Central Macular** | 274.24±17.22 | 276.38±19.11 | 276.00 (258.00;287.00) | 274.00 (264.00;290.00) | -0.776 |
| **Outer N** | 313.89±14.19 | 311.76±12.02 | 317.00 (303.00;323.00) | 313.00 (305.00;321.00) | 0.683 |
| **Inner N** | 341.43±13.75 | 343.65±12.26 | 342.00 (334.00;352.00) | 344.00 (337.00;351.00) | -0.647 |
| **Outer S** | 298.54±11.10 | 297.70±11.52 | 299.00 (289.00;306.00) | 298.00 (292.00;306.00) | 0.281 |
| **Inner S** | 341.24±12.34 | 341.30±12.29 | 342.00 (333.00;351.00) | 340.00 (331.00;352.00) | -0.016 |
| **Outer I** | 287.05±10.73 | 289.97±12.29 | 285.00 (279.00;296.00) | 290.00 (280.00;295.00) | -1.012 |
| **Inner I** | 337.54±10.88 | 337.59±13.38 | 337.00 (329.00;344.00) | 339.00 (330.00;347.00) | -0.016 |
| **Central Retinal Thickness** | 234.24±20.26 | 236.65±25.81 | 232.00 (221.00;248.00) | 231.00 (218.00;243.00) | -1.022 |
| **Total retinal volume (mm³)** | | | | | |
| **Outer T** | 1.49±0.06 | 1.50±0.06 | 1.49 (1.45;1.54) | 1.50 (1.45;1.55) | -0.794 |
| **Inner T** | 0.51±0.02 | 0.51±0.02 | 0.51 (0.50;0.53) | 0.51 (0.50;0.53) | -0.211 |
| **Central Macular** | 0.22±0.01 | 0.22±0.02 | 0.22 (0.20;0.23) | 0.22 (0.21;0.23) | -0.374 |
| **Outer N** | 1.66±0.07 | 1.65±0.06 | 1.66 (1.61;1.70) | 1.66 (1.61;1.70) | 0.278 |
| **Inner N** | 0.54±0.02 | 0.54±0.02 | 0.54 (0.53;0.55) | 0.54 (0.53;0.55) | -0.251 |
| **Outer S** | 1.58±0.06 | 1.58±0.06 | 1.59 (1.53;1.62) | 1.58 (1.55;1.62) | 0.188 |
| **Inner S** | 0.54±0.02 | 0.54±0.02 | 0.54 (0.52;0.55) | 0.54 (0.52;0.55) | 0.390 |
| **Outer I** | 1.52±0.06 | 1.54±0.07 | 1.51 (1.48;1.57) | 1.53 (1.48;1.56) | -0.884 |
| **Inner I** | 0.53±0.02 | 0.53±0.02 | 0.53 (0.52;0.54) | 0.53 (0.52;0.55) | 0.051 |
| **Total** | 8.59±0.28 | 8.55±0.44 | 8.60 (8.32;8.81) | 8.60 (8.31;8.80) | 0.397 |
| **ORL thickness (μm)** | | | | | |
| **Inner T** | 81.41±2.20 | 81.57±2.52 | 82.00 (80.00;83.00) | 82.00 (80.00;83.00) | -0.199 |
| **Central Macular** | 88.59±3.01 | 88.30±2.67 | 89.00 (87.00;91.00) | 88.00 (87.00;89.00) | 0.336 |
| **Inner N** | 82.54±2.56 | 82.08±2.36 | 82.00 (81.00;84.00) | 82.00 (81.00;84.00) | 0.558 |
| **Inner S** | 81.43±2.43 | 81.49±2.45 | 82.00 (81.00;83.00) | 81.00 (80.00;83.00) | -0.066 |
| **Inner I** | 79.97±2.72 | 80.41±3.05 | 80.00 (78.00;82.00) | 80.00 (78.00;82.00) | -0.539 |
| **RPE thickness (μm)** | | | | | |
| **Inner T** | 14.30±0.78 | 14.43±0.83 | 14.00 (14.00;15.00) | 14.00 (14.00;15.00) | -0.941 |
| **Central Macular** | 16.03±1.46 | 15.65±1.27 | 16.00 (15.00;17.00) | 16.00 (15.00;16.00) | 2.389 |
| **Inner N** | 15.46±1.24 | 15.03±0.93 | 15.00 (15.00;16.00) | 15.00 (14.00;15.00) | 2.837 |
| **Inner S** | 15.46±1.48 | 15.43±1.01 | 15.00 (14.00;16.00) | 15.00 (15.00;16.00) | 0.175 |
| **Inner I** | 14.70±1.20 | 14.89±1.17 | 15.00 (14.00;15.00) | 15.00 (14.00;15.00) | -1.279 |
| **ONL thickness (μm)** | | | | | |
| **Inner T** | 69.95±9.99 | 72.86±8.23 | 72.00 (64.00;76.00) | 73.00 (67.00;79.00) | -4.088 |
| **Central Macular** | 91.51±11.20 | 91.59±12.09 | 93.00 (84.00;99.00) | 94.00 (87.00;100.00) | -0.089 |
| **Inner N** | 72.73±9.29 | 68.84±11.18 | 70.00 (66.00;79.00) | 71.00 (63.00;75.00) | 5.498 |
| **Inner S** | 67.14±10.63 | 67.81±10.59 | 70.00 (60.00;75.00) | 69.00 (61.00;76.00) | -1.001 |
| **Inner I** | 67.62±10.95 | 65.73±11.20 | 70.00 (60.00;74.00) | 67.00 (61.00;73.00) | 2.837 |
| **Choroidal thickness (μm)** | | | | | |
| **Outer T** | 262.08±57.89 | 277.84±64.05 | 267.00 (218.00;281.00) | 269.00 (239.00;322.00) | -5.837 |
| **Inner T** | 301.16±70.66 | 305.59±80.72 | 299.00 (244.00;351.00) | 303.00 (253.00;347.00) | -1.461 |
| **Central Macular** | 324.27±74.15 | 316.08±92.63 | 326.00 (273.00;373.00) | 313.00 (261.00;380.00) | 2.558 |
| **Outer N** | 248.16±63.74 | 239.24±68.00 | 249.00 (202.00;283.00) | 225.00 (194.00;282.00) | 3.660 |
| **Inner N** | 302.89±82.33 | 301.44±87.46 | 313.00 (244.00;351.00) | 297.00 (251.75;349.50) | 0.479 |
| **Outer S** | 304.76±67.70 | 318.65±69.11 | 313.00 (249.00;339.00) | 303.00 (276.00;346.00) | -4.457 |
| **Inner S** | 322.16±77.00 | 328.38±80.61 | 328.00 (266.00;374.00) | 325.00 (266.00;378.00) | -1.911 |
| **Outer I** | 270.57±69.08 | 272.30±81.53 | 287.00 (214.00;319.00) | 257.00 (215.00;328.00) | -0.637 |
| **Inner I** | 300.73±74.90 | 292.41±90.17 | 308.00 (247.00;346.00) | 271.00 (222.00;352.00) | 2.807 |
| **SFCT** | 323.97±75.58 | 318.05±103.43 | 323.00 (269.00;387.00) | 304.00 (259.00;395.00) | 1.844 |
| **Choroidal volume (mm³)** | | | | | |
| **Outer T** | 1.38±0.29 | 1.47±0.34 | 1.41 (1.16;1.50) | 1.42 (1.26;1.71) | -6.521 |
| **Inner T** | 0.47±0.11 | 0.48±0.13 | 0.47 (0.38;0.55) | 0.48 (0.40;0.55) | -1.304 |
| **Central Macular** | 0.25±0.06 | 0.25±0.07 | 0.26 (0.21;0.29) | 0.24 (0.20;0.30) | 2.148 |
| **Outer N** | 1.30±0.35 | 1.25±0.37 | 1.31 (1.07;1.47) | 1.17 (1.02;1.50) | 3.544 |
| **Inner N** | 0.48±0.12 | 0.47±0.14 | 0.49 (0.40;0.55) | 0.46 (0.39;0.54) | 2.844 |
| **Outer S** | 1.62±0.36 | 1.70±0.37 | 1.66 (1.32;1.80) | 1.66 (1.47;1.96) | -4.806 |
| **Inner S** | 0.51±0.12 | 0.52±0.13 | 0.52 (0.42;0.58) | 0.51 (0.42;0.59) | -2.011 |
| **Outer I** | 1.43±0.37 | 1.41±0.42 | 1.52 (1.13;1.69) | 1.30 (1.13;1.73) | 1.885 |
| **Inner I** | 0.47±0.12 | 0.46±0.14 | 0.48 (0.39;0.54) | 0.46 (0.35;0.56) | 2.082 |
| **Total** | 7.91±1.71 | 8.03±1.90 | 8.03 (6.69;8.90) | 7.95 (6.69;9.05) | -1.532 |
| **Other choroidal parameters** | | | | | |
| **mTCA (μm²)** | 359 412.07±81 861.65 | 358 257.23±96 498.77 | 359479.00 (303 612.50;418 928.00) | 362686.00 (283 990.50;412 109.00) | 0.322 |
| **mLA (μm²)** | 238 211.19±49 757.14 | 236 178.22±62 787.28 | 239348.00 (199 758.00;269 454.00) | 248209.00 (191 325.50;271 393.00) | 0.857 |
| **mSA (μm²)** | 121 200.88±33 916.92 | 122 079.01±35 742.59 | 119187.50 (98 616.00;144 139.50) | 117603.00 (96 765.00;149 759.00) | -0.722 |
| **mCVI (%)** | 66.61±3.04 | 65.98±2.64 | 66.00 (64.31;69.43) | 65.63 (64.27;67.09) | 0.957 |

Relative mean difference (%) was calculated as the percent difference between left and right eyes normalized to their mean:

$$\text{Relative mean difference (\%) }=\frac{\text{Left - Right}}{(\text{Left + Right})/2}\times100$$

ETDRS subfields refer to the conventional Early Treatment Diabetic Retinopathy Study grid centered on the fovea: Central Macular – central subfield (within a 500-μm radius); Inner – inner ring (500–1500 μm); Outer – outer ring (1500–3000 μm). Thus “Inner T” denotes the temporal subfield of the inner ring. “Inner” and “Outer” denote topographic ETDRS rings and not retinal depth; retinal layers are designated separately as ONL, ORL and RPE.

Abbreviations: CVI – choroidal vascularity index; I – inferior; mCVI – macular choroidal vascularity index; mLA – macular luminal area; mSA – macular stromal area; mTCA – macular total choroidal area; N – nasal; OCT – optical coherence tomography; ONL – outer nuclear layer; ORL – outer retinal layer; Q1 – first quartile; Q3 – third quartile; RPE – retinal pigment epithelium; SD – standard deviation; SFCT – subfoveal choroidal thickness; S – superior; T – temporal.

**Table S4. Disease-related inter-eye asymmetry of retinal and choroidal OCT parameters in patients with precapillary pulmonary hypertension versus healthy controls.**

| **Variable** | **Absolute difference between left and right eyes - PH group (mean ± SD)** | **Absolute difference between left and right eyes - control group (mean ± SD)** | **Absolute difference between left and right eyes - PH group (median [Q1; Q3])** | **Absolute difference between left and right eyes - control group (median [Q1; Q3])** | **p-value of Mann–Whitney U test (w/o correction) between PH and control groups** | **p-value of Mann–Whitney U test (BH correction) between PH and control groups** |
| --- | --- | --- | --- | --- | --- | --- |
| **Total retinal thickness (μm)** | | | | | | |
| **Outer T** | 14.82±15.57 | 4.73±4.78 | 9.00 (1.75;23.00) | 4.00 (1.00;6.00) | 0.039 | 0.098 |
| **Inner T** | 13.39±11.55 | 3.14±3.03 | 10.50 (4.00;19.00) | 2.00 (1.00;5.00) | <0.001 | <0.001 |
| **Central Macular** | 14.96±20.67 | 6.89±9.99 | 7.00 (2.00;19.50) | 4.00 (1.00;9.00) | 0.106 | 0.177 |
| **Outer N** | 16.82±19.61 | 5.05±6.77 | 7.00 (4.00;20.50) | 3.00 (2.00;5.00) | <0.001 | <0.001 |
| **Inner N** | 14.39±15.04 | 4.81±6.33 | 10.00 (3.75;19.00) | 3.00 (1.00;5.00) | 0.001 | 0.003 |
| **Outer S** | 10.07±17.98 | 5.11±4.03 | 5.00 (2.75;9.25) | 4.00 (2.00;7.00) | 0.436 | 0.484 |
| **Inner S** | 10.68±21.33 | 4.11±3.79 | 5.00 (2.00;9.25) | 3.00 (1.00;6.00) | 0.103 | 0.177 |
| **Outer I** | 15.68±28.79 | 5.51±5.71 | 7.50 (1.75;15.25) | 3.00 (2.00;8.00) | 0.239 | 0.299 |
| **Inner I** | 7.71±7.47 | 5.19±5.88 | 5.50 (2.00;10.50) | 3.00 (2.00;6.00) | 0.226 | 0.299 |
| **Central Retinal Thickness** | 18.50±27.17 | 13.16±17.48 | 8.50 (3.75;15.75) | 6.00 (3.00;15.00) | 0.638 | 0.638 |
| **Total retinal volume (mm³)** | | | | | | |
| **Outer T** | 0.10±0.09 | 0.02±0.02 | 0.09 (0.01;0.18) | 0.02 (0.01;0.03) | 0.015 | 0.038 |
| **Inner T** | 0.03±0.03 | 0.00±0.01 | 0.02 (0.01;0.03) | 0.00 (0.00;0.01) | <0.001 | <0.001 |
| **Central Macular** | 0.01±0.01 | 0.01±0.01 | 0.01 (0.00;0.01) | 0.00 (0.00;0.01) | 0.165 | 0.206 |
| **Outer N** | 0.07±0.06 | 0.02±0.01 | 0.04 (0.02;0.11) | 0.02 (0.01;0.03) | <0.001 | <0.001 |
| **Inner N** | 0.02±0.02 | 0.01±0.01 | 0.02 (0.01;0.03) | 0.01 (0.00;0.01) | 0.001 | 0.003 |
| **Outer S** | 0.04±0.04 | 0.03±0.02 | 0.03 (0.02;0.04) | 0.02 (0.01;0.03) | 0.323 | 0.323 |
| **Inner S** | 0.01±0.01 | 0.01±0.01 | 0.01 (0.01;0.01) | 0.01 (0.00;0.01) | 0.052 | 0.087 |
| **Outer I** | 0.06±0.13 | 0.03±0.03 | 0.03 (0.01;0.07) | 0.02 (0.01;0.04) | 0.254 | 0.282 |
| **Inner I** | 0.01±0.01 | 0.01±0.01 | 0.01 (0.00;0.01) | 0.00 (0.00;0.01) | 0.158 | 0.206 |
| **Total** | 0.18±0.19 | 0.12±0.34 | 0.11 (0.05;0.26) | 0.05 (0.03;0.09) | 0.025 | 0.050 |
| **ORL thickness (μm)** | | | | | | |
| **Inner T** | 1.46±0.96 | 1.73±1.39 | 1.00 (1.00;2.00) | 1.00 (1.00;2.00) | 0.688 | 0.688 |
| **Central Macular** | 2.61±2.17 | 2.14±1.77 | 2.00 (1.00;4.00) | 2.00 (1.00;3.00) | 0.429 | 0.536 |
| **Inner N** | 1.79±1.23 | 1.38±1.14 | 2.00 (1.00;3.00) | 1.00 (1.00;2.00) | 0.168 | 0.345 |
| **Inner S** | 1.25±1.27 | 1.57±1.30 | 1.00 (1.00;1.00) | 1.00 (1.00;2.00) | 0.157 | 0.345 |
| **Inner I** | 1.89±1.59 | 1.41±1.32 | 2.00 (1.00;3.00) | 1.00 (0.00;2.00) | 0.207 | 0.345 |
| **RPE thickness (μm)** | | | | | | |
| **Inner T** | 0.82±0.72 | 0.68±0.63 | 1.00 (0.00;1.00) | 1.00 (0.00;1.00) | 0.443 | 0.738 |
| **Central Macular** | 0.96±0.96 | 1.30±1.08 | 1.00 (0.00;1.25) | 1.00 (0.00;2.00) | 0.212 | 0.730 |
| **Inner N** | 0.93±0.81 | 1.03±0.83 | 1.00 (0.00;1.00) | 1.00 (0.00;1.00) | 0.648 | 0.810 |
| **Inner S** | 0.89±0.96 | 1.05±0.81 | 1.00 (0.00;1.00) | 1.00 (0.00;2.00) | 0.292 | 0.730 |
| **Inner I** | 0.93±0.72 | 0.95±0.78 | 1.00 (0.75;1.00) | 1.00 (0.00;1.00) | 0.942 | 0.942 |
| **ONL thickness (μm)** | | | | | | |
| **Inner T** | 5.89±4.60 | 4.92±5.49 | 4.00 (3.00;8.00) | 3.00 (2.00;6.00) | 0.124 | 0.473 |
| **Central Macular** | 7.75±7.38 | 4.89±5.26 | 5.50 (1.00;13.25) | 3.00 (2.00;7.00) | 0.284 | 0.473 |
| **Inner N** | 7.71±7.90 | 6.54±6.79 | 4.50 (2.00;12.25) | 3.00 (2.00;11.00) | 0.536 | 0.536 |
| **Inner S** | 9.43±8.15 | 6.89±5.90 | 7.50 (3.00;14.25) | 4.00 (2.00;12.00) | 0.247 | 0.473 |
| **Inner I** | 6.89±6.51 | 5.08±4.84 | 4.50 (2.00;8.50) | 4.00 (2.00;6.00) | 0.398 | 0.498 |
| **Choroidal thickness (μm)** | | | | | | |
| **Outer T** | 33.86±40.05 | 32.84±25.98 | 26.00 (12.25;41.00) | 29.00 (9.00;44.00) | 0.667 | 0.786 |
| **Inner T** | 38.71±39.78 | 38.11±30.07 | 25.00 (18.00;39.75) | 34.00 (15.00;55.00) | 0.786 | 0.786 |
| **Central Macular** | 44.68±43.15 | 44.89±33.93 | 32.00 (14.00;60.25) | 40.00 (23.00;65.00) | 0.624 | 0.786 |
| **Outer N** | 32.71±26.73 | 38.38±33.09 | 25.00 (14.00;43.50) | 27.00 (15.00;55.00) | 0.726 | 0.786 |
| **Inner N** | 46.64±44.95 | 36.97±34.93 | 28.00 (10.50;73.75) | 35.00 (7.50;52.75) | 0.444 | 0.786 |
| **Outer S** | 37.29±31.87 | 33.68±30.22 | 30.50 (16.00;47.00) | 23.00 (16.00;40.00) | 0.538 | 0.786 |
| **Inner S** | 45.79±50.10 | 40.81±31.54 | 25.50 (17.00;61.00) | 33.00 (17.00;55.00) | 0.786 | 0.786 |
| **Outer I** | 37.26±44.43 | 38.22±32.30 | 20.00 (6.50;46.00) | 26.00 (17.00;55.00) | 0.314 | 0.786 |
| **Inner I** | 29.64±33.19 | 46.16±40.42 | 19.50 (6.75;33.00) | 38.00 (18.00;63.00) | 0.056 | 0.560 |
| **SFCT** | 52.71±44.94 | 59.05±45.80 | 38.50 (19.50;70.50) | 40.00 (27.00;89.00) | 0.427 | 0.786 |
| **Choroidal volume (mm³)** | | | | | | |
| **Outer T** | 0.19±0.22 | 0.18±0.14 | 0.16 (0.07;0.23) | 0.18 (0.05;0.24) | 0.623 | 0.894 |
| **Inner T** | 0.07±0.06 | 0.06±0.05 | 0.04 (0.03;0.07) | 0.06 (0.02;0.09) | 0.894 | 0.894 |
| **Central Macular** | 0.03±0.03 | 0.04±0.03 | 0.03 (0.01;0.05) | 0.03 (0.02;0.05) | 0.487 | 0.894 |
| **Outer N** | 0.22±0.24 | 0.16±0.14 | 0.15 (0.09;0.29) | 0.10 (0.06;0.22) | 0.347 | 0.894 |
| **Inner N** | 0.09±0.18 | 0.06±0.06 | 0.04 (0.02;0.07) | 0.06 (0.01;0.08) | 0.894 | 0.894 |
| **Outer S** | 0.22±0.26 | 0.18±0.16 | 0.14 (0.09;0.23) | 0.13 (0.08;0.21) | 0.662 | 0.894 |
| **Inner S** | 0.07±0.07 | 0.06±0.05 | 0.04 (0.02;0.09) | 0.05 (0.02;0.08) | 0.613 | 0.894 |
| **Outer I** | 0.18±0.21 | 0.21±0.18 | 0.12 (0.03;0.23) | 0.13 (0.09;0.29) | 0.262 | 0.894 |
| **Inner I** | 0.05±0.05 | 0.07±0.06 | 0.03 (0.01;0.06) | 0.05 (0.03;0.09) | 0.056 | 0.560 |
| **Total** | 1.03±1.84 | 0.73±0.56 | 0.50 (0.32;0.83) | 0.49 (0.31;1.12) | 0.806 | 0.894 |
| **Other choroidal parameters** | | | | | | |
| **mTCA (μm²)** | 38 321.37±33 777.32 | 46 306.76±34 160.45 | 32168.00 (15 274.25;52 920.50) | 39871.50 (25 393.50;58 869.00) | 0.269 | 0.359 |
| **mLA (μm²)** | 24 776.02±21 054.86 | 30 814.35±21 251.57 | 18255.00 (9 216.25;35 008.25) | 25126.00 (13 444.00;46 319.50) | 0.209 | 0.359 |
| **mSA (μm²)** | 15 309.83±13 198.91 | 17 665.05±14 338.45 | 9358.50 (5 986.75;19 125.75) | 15503.00 (6 958.00;21 034.00) | 0.423 | 0.423 |
| **mCVI (%)** | 1.37±1.05 | 2.18±1.58 | 1.19 (0.62;1.55) | 1.86 (1.07;2.96) | 0.031 | 0.124 |

Benjamini–Hochberg correction was applied separately for each group of parameters (i.e., total retinal thickness, total retinal volume, ORL thickness, RPE thickness, ONL thickness, choroidal thickness, choroidal volume, and other choroidal parameters).

The absolute interocular difference was calculated as the absolute value of the difference between left and right eyes:

$$\text{Absolute interocular difference }=\mid\text{Left - Right}\mid$$

ETDRS subfields refer to the conventional Early Treatment Diabetic Retinopathy Study grid centered on the fovea: Central Macular – central subfield (within a 500-μm radius); Inner – inner ring (500–1500 μm); Outer – outer ring (1500–3000 μm). Thus “Inner T” denotes the temporal subfield of the inner ring. “Inner” and “Outer” denote topographic ETDRS rings and not retinal depth; retinal layers are designated separately as ONL, ORL and RPE.

Abbreviations: BH – Benjamini–Hochberg; CVI – choroidal vascularity index; I – inferior; mCVI – macular choroidal vascularity index; mLA – macular luminal area; mSA – macular stromal area; mTCA – macular total choroidal area; N – nasal; OCT – optical coherence tomography; ONL – outer nuclear layer; ORL – outer retinal layer; PH – pulmonary hypertension; Q1 – first quartile; Q3 – third quartile; RPE – retinal pigment epithelium; SD – standard deviation; SFCT – subfoveal choroidal thickness; S – superior; T – temporal.

**Table S5. Associations of mean arterial pressure and caffeine consumption with absolute interocular asymmetry in the overall study population.**

| **Variable** | **MAP** | | **Caffeine** | |
| --- | --- | --- | --- | --- |
|  | **β (SE)** | **p-value (BH correction)** | **β (SE)** | **p-value (BH correction)** |
| **Total retinal thickness (μm)** | | | | |
| **Outer T** | 0.036 (0.041) | 0.639 | 1.125 (1.274) | 0.655 |
| **Inner T** | 0.027 (0.026) | 0.639 | -1.103 (0.747) | 0.618 |
| **Central Macular** | 0.037 (0.073) | 0.876 | 1.319 (2.058) | 0.655 |
| **Outer N** | 0.054 (0.058) | 0.639 | 0.665 (1.790) | 0.712 |
| **Inner N** | 0.004 (0.051) | 0.943 | 1.536 (1.128) | 0.618 |
| **Outer S** | -0.033 (0.031) | 0.639 | 2.627 (1.959) | 0.618 |
| **Inner S** | -0.008 (0.027) | 0.879 | 1.445 (2.065) | 0.655 |
| **Outer I** | 0.143 (0.127) | 0.639 | 0.869 (2.252) | 0.712 |
| **Inner I** | -0.041 (0.026) | 0.639 | -0.329 (0.503) | 0.655 |
| **Central Retinal Thickness** | -0.033 (0.123) | 0.879 | -1.986 (2.514) | 0.655 |
| **Total retinal volume (mm³)** | | | | |
| **Outer T** | 0.014 (0.048) | 0.885 | 0.135 (1.322) | 0.960 |
| **Inner T** | 0.005 (0.032) | 0.885 | -0.072 (1.440) | 0.960 |
| **Central Macular** | -0.019 (0.075) | 0.885 | 0.342 (1.233) | 0.960 |
| **Outer N** | 0.037 (0.029) | 0.850 | -1.052 (0.837) | 0.879 |
| **Inner N** | 0.038 (0.033) | 0.850 | 1.239 (1.117) | 0.879 |
| **Outer S** | -0.015 (0.023) | 0.868 | 0.815 (0.678) | 0.879 |
| **Inner S** | -0.018 (0.020) | 0.850 | -0.060 (0.618) | 0.960 |
| **Outer I** | 0.100 (0.125) | 0.850 | -0.775 (1.169) | 0.960 |
| **Inner I** | -0.045 (0.032) | 0.850 | -0.096 (0.549) | 0.960 |
| **Total** | 0.013 (0.028) | 0.885 | -1.472 (1.566) | 0.879 |
| **ORL thickness (μm)** | | | | |
| **Inner T** | -0.008 (0.018) | 0.811 | -0.404 (0.476) | 0.460 |
| **Central Macular** | -0.022 (0.040) | 0.811 | -0.457 (0.614) | 0.460 |
| **Inner N** | 0.001 (0.020) | 0.974 | -0.879 (0.451) | 0.282 |
| **Inner S** | -0.016 (0.019) | 0.811 | -0.695 (0.500) | 0.426 |
| **Inner I** | -0.023 (0.025) | 0.811 | -0.429 (0.528) | 0.460 |
| **RPE thickness (μm)** | | | | |
| **Inner T** | 0.026 (0.063) | 0.794 | -1.865 (1.360) | 0.368 |
| **Central Macular** | -0.067 (0.102) | 0.794 | -1.396 (2.049) | 0.499 |
| **Inner N** | -0.014 (0.053) | 0.794 | -1.894 (1.530) | 0.368 |
| **Inner S** | 0.037 (0.078) | 0.794 | -1.377 (1.701) | 0.499 |
| **Inner I** | -0.037 (0.070) | 0.794 | -3.052 (1.291) | 0.108 |
| **ONL thickness (μm)** | | | | |
| **Inner T** | -0.053 (0.133) | 0.919 | -0.332 (2.328) | 0.887 |
| **Central Macular** | 0.005 (0.100) | 0.962 | 1.634 (2.284) | 0.597 |
| **Inner N** | 0.092 (0.272) | 0.919 | 3.711 (3.413) | 0.469 |
| **Inner S** | 0.078 (0.147) | 0.919 | -4.004 (3.157) | 0.469 |
| **Inner I** | 0.249 (0.248) | 0.919 | -3.838 (2.782) | 0.469 |
| **Choroidal thickness (μm)** | | | | |
| **Outer T** | -0.121 (0.163) | 0.605 | 5.509 (4.248) | 0.488 |
| **Inner T** | 0.050 (0.206) | 0.811 | 4.007 (4.632) | 0.488 |
| **Central Macular** | 0.189 (0.235) | 0.605 | 5.368 (5.194) | 0.488 |
| **Outer N** | 0.157 (0.198) | 0.605 | 5.063 (4.105) | 0.488 |
| **Inner N** | 0.169 (0.239) | 0.605 | 3.622 (5.387) | 0.560 |
| **Outer S** | 0.087 (0.218) | 0.768 | -3.194 (3.280) | 0.488 |
| **Inner S** | 0.250 (0.305) | 0.605 | 7.210 (6.490) | 0.488 |
| **Outer I** | 0.536 (0.167) | 0.022 | 4.746 (5.452) | 0.488 |
| **Inner I** | 0.468 (0.219) | 0.184 | 5.942 (5.166) | 0.488 |
| **SFCT** | 0.243 (0.286) | 0.605 | -0.614 (4.687) | 0.896 |
| **Choroidal volume (mm³)** | | | | |
| **Outer T** | -0.146 (0.176) | 0.722 | 5.515 (4.230) | 0.494 |
| **Inner T** | 0.064 (0.210) | 0.847 | 4.390 (4.669) | 0.525 |
| **Central Macular** | 0.149 (0.233) | 0.722 | 4.526 (4.447) | 0.525 |
| **Outer N** | 0.194 (0.221) | 0.722 | 8.307 (4.639) | 0.394 |
| **Inner N** | 0.255 (0.216) | 0.722 | 6.107 (5.942) | 0.525 |
| **Outer S** | 0.038 (0.223) | 0.866 | -6.144 (3.371) | 0.394 |
| **Inner S** | 0.238 (0.309) | 0.722 | 4.216 (4.968) | 0.525 |
| **Outer I** | 0.496 (0.179) | 0.077 | 3.134 (4.829) | 0.577 |
| **Inner I** | 0.431 (0.234) | 0.354 | 7.411 (5.035) | 0.489 |
| **Total** | -0.105 (0.188) | 0.722 | -1.682 (5.186) | 0.747 |
| **Other choroidal parameters** | | | | |
| **mTCA (μm²)** | 0.063 (0.150) | 0.899 | 1.016 (3.108) | 0.861 |
| **mLA (μm²)** | -0.014 (0.124) | 0.912 | -0.564 (3.209) | 0.861 |
| **mSA (μm²)** | 0.156 (0.191) | 0.840 | 2.054 (3.158) | 0.861 |
| **mCVI (%)** | 0.036 (0.031) | 0.840 | -0.832 (0.724) | 0.861 |

Absolute interocular asymmetry was calculated as the absolute percent difference between left and right eyes normalized to their mean, with higher values indicating greater asymmetry between eyes:

$$\text{Absolute interocular asymmetry }=\frac{\text{|Left - Right|}}{(\text{Left + Right})/2}\times100$$

Each row indicates one multivariable linear regression model with absolute interocular asymmetry variable as response variable (1^st^ column). Predictor variables included into each model were: study group (precapillary PH vs. control), mean arterial pressure (MAP), and caffeine consumption. Regression coefficients (β) with HC3 heteroskedasticity-robust standard errors (SE) are presented. For continuous MAP, β represents the change in absolute asymmetry, in percentage points, per 1-mmHg increase in MAP. For caffeine consumption, positive and negative β values indicate greater and lower asymmetry, respectively, relative to participants reporting no caffeine consumption. Benjamini–Hochberg correction was applied separately for each group of parameters (i.e., total retinal thickness, total retinal volume, ORL thickness, RPE thickness, ONL thickness, choroidal thickness, choroidal volume, and other choroidal parameters), and separately for each predictor.

ETDRS subfields refer to the conventional Early Treatment Diabetic Retinopathy Study grid centered on the fovea: Central Macular – central subfield (within a 500-μm radius); Inner – inner ring (500–1500 μm); Outer – outer ring (1500–3000 μm). Thus “Inner T” denotes the temporal subfield of the inner ring. “Inner” and “Outer” denote topographic ETDRS rings and not retinal depth; retinal layers are designated separately as ONL, ORL and RPE.

Abbreviations: BH – Benjamini–Hochberg; CVI – choroidal vascularity index; HC3 – heteroskedasticity-consistent standard errors (type 3); I – inferior; MAP – mean arterial pressure; mCVI – macular choroidal vascularity index; mLA – macular luminal area; mSA – macular stromal area; mTCA – macular total choroidal area; N – nasal; OCT – optical coherence tomography; ONL – outer nuclear layer; ORL – outer retinal layer; PH – pulmonary hypertension; RPE – retinal pigment epithelium; SE – standard error; SFCT – subfoveal choroidal thickness; S – superior; T – temporal.

**Table S6. Associations of mean arterial pressure, caffeine consumption, and sildenafil use with absolute interocular asymmetry in patients with precapillary pulmonary hypertension.**

| **Variable** | **MAP** | | **Caffeine** | | **Sildenafil** | |
| --- | --- | --- | --- | --- | --- | --- |
|  | **β (SE)** | **p-value (BH correction)** | **β (SE)** | **p-value (BH correction)** | **β (SE)** | **p-value (BH correction)** |
| **Total retinal thickness (μm)** | | | | | | |
| **Outer T** | 0.014 (0.115) | 0.906 | 2.038 (2.429) | 0.664 | 0.137 (2.319) | 0.980 |
| **Inner T** | 0.099 (0.067) | 0.714 | -2.003 (1.096) | 0.531 | -2.605 (1.481) | 0.924 |
| **Central Macular** | 0.233 (0.172) | 0.714 | 2.193 (5.042) | 0.668 | -3.247 (4.641) | 0.951 |
| **Outer N** | 0.201 (0.157) | 0.714 | -1.146 (2.175) | 0.668 | -0.935 (2.229) | 0.951 |
| **Inner N** | 0.095 (0.164) | 0.809 | 2.979 (2.495) | 0.664 | -0.051 (2.036) | 0.980 |
| **Outer S** | -0.104 (0.111) | 0.722 | 4.620 (4.065) | 0.664 | 3.386 (2.603) | 0.951 |
| **Inner S** | 0.046 (0.118) | 0.862 | 4.659 (5.288) | 0.664 | -4.596 (4.684) | 0.951 |
| **Outer I** | 0.497 (0.456) | 0.718 | 3.004 (4.916) | 0.668 | -1.632 (5.287) | 0.951 |
| **Inner I** | -0.035 (0.043) | 0.722 | -0.732 (0.983) | 0.664 | -0.424 (1.150) | 0.951 |
| **Central Retinal Thickness** | -0.086 (0.297) | 0.862 | -6.253 (3.712) | 0.531 | 2.891 (4.645) | 0.951 |
| **Total retinal volume (mm³)** | | | | | | |
| **Outer T** | -0.047 (0.157) | 0.851 | 0.062 (2.746) | 0.982 | 1.181 (2.708) | 0.845 |
| **Inner T** | 0.061 (0.101) | 0.792 | 0.651 (3.225) | 0.982 | -5.465 (2.998) | 0.410 |
| **Central Macular** | 0.017 (0.175) | 0.925 | -1.079 (2.133) | 0.982 | 0.789 (2.558) | 0.845 |
| **Outer N** | 0.134 (0.076) | 0.459 | -1.180 (1.714) | 0.982 | -2.638 (1.830) | 0.545 |
| **Inner N** | 0.161 (0.053) | 0.063 | 1.976 (1.395) | 0.982 | -2.624 (1.185) | 0.374 |
| **Outer S** | -0.033 (0.041) | 0.758 | 0.697 (1.289) | 0.982 | 1.257 (1.070) | 0.631 |
| **Inner S** | -0.033 (0.041) | 0.758 | 0.163 (1.082) | 0.982 | 0.346 (1.050) | 0.845 |
| **Outer I** | 0.359 (0.471) | 0.758 | -0.841 (2.479) | 0.982 | -3.570 (5.276) | 0.845 |
| **Inner I** | -0.057 (0.045) | 0.711 | -0.306 (1.153) | 0.982 | -0.658 (1.295) | 0.845 |
| **Total** | -0.027 (0.071) | 0.851 | -0.082 (1.158) | 0.982 | 0.026 (1.225) | 0.984 |
| **ORL thickness (μm)** | | | | | | |
| **Inner T** | -0.036 (0.028) | 0.523 | 0.152 (0.517) | 0.771 | 0.586 (0.564) | 0.776 |
| **Central Macular** | 0.062 (0.082) | 0.721 | -1.088 (1.379) | 0.548 | -0.208 (1.451) | 0.989 |
| **Inner N** | 0.061 (0.043) | 0.523 | -0.754 (0.726) | 0.517 | -0.009 (0.641) | 0.989 |
| **Inner S** | 0.019 (0.034) | 0.721 | -0.809 (0.546) | 0.382 | -0.970 (0.797) | 0.776 |
| **Inner I** | 0.012 (0.070) | 0.860 | -1.688 (0.871) | 0.328 | 0.407 (0.987) | 0.989 |
| **RPE thickness (μm)** | | | | | | |
| **Inner T** | 0.146 (0.171) | 0.822 | -0.831 (2.495) | 0.909 | -0.418 (2.742) | 0.880 |
| **Central Macular** | 0.089 (0.223) | 0.822 | -0.322 (2.779) | 0.909 | 1.548 (3.098) | 0.778 |
| **Inner N** | 0.092 (0.162) | 0.822 | -0.980 (3.307) | 0.909 | -3.754 (3.080) | 0.589 |
| **Inner S** | 0.050 (0.221) | 0.822 | -2.279 (3.022) | 0.909 | 2.046 (3.251) | 0.778 |
| **Inner I** | 0.096 (0.103) | 0.822 | -3.080 (2.141) | 0.822 | 2.554 (1.943) | 0.589 |
| **ONL thickness (μm)** | | | | | | |
| **Inner T** | 0.012 (0.143) | 0.936 | -0.910 (3.297) | 0.981 | -1.954 (3.417) | 0.760 |
| **Central Macular** | 0.033 (0.278) | 0.936 | -0.035 (5.018) | 0.994 | -4.004 (5.144) | 0.760 |
| **Inner N** | 0.662 (0.583) | 0.936 | 4.783 (6.348) | 0.765 | -8.402 (7.737) | 0.760 |
| **Inner S** | -0.116 (0.277) | 0.936 | -9.027 (5.769) | 0.330 | -1.834 (5.925) | 0.760 |
| **Inner I** | 0.073 (0.354) | 0.936 | -11.006 (3.870) | 0.047 | -2.881 (5.552) | 0.760 |
| **Choroidal thickness (μm)** | | | | | | |
| **Outer T** | 0.077 (0.437) | 0.950 | 8.064 (8.390) | 0.687 | 2.044 (6.345) | 0.834 |
| **Inner T** | 0.243 (0.574) | 0.846 | 6.333 (8.830) | 0.687 | 5.657 (7.606) | 0.834 |
| **Central Macular** | 0.478 (0.777) | 0.846 | 11.859 (11.501) | 0.687 | -2.330 (10.560) | 0.834 |
| **Outer N** | 0.025 (0.389) | 0.950 | 6.603 (8.325) | 0.687 | 2.963 (7.856) | 0.834 |
| **Inner N** | 0.355 (0.560) | 0.846 | 5.980 (12.155) | 0.757 | 4.040 (10.554) | 0.834 |
| **Outer S** | 0.539 (0.684) | 0.846 | -2.378 (5.712) | 0.757 | 2.437 (6.672) | 0.834 |
| **Inner S** | 1.541 (0.579) | 0.143 | 13.661 (14.984) | 0.687 | -9.069 (11.164) | 0.834 |
| **Outer I** | 0.987 (0.516) | 0.348 | 11.406 (10.224) | 0.687 | 1.589 (7.496) | 0.834 |
| **Inner I** | 0.151 (0.352) | 0.846 | 8.310 (7.458) | 0.687 | 5.617 (5.473) | 0.834 |
| **SFCT** | 0.997 (0.645) | 0.455 | -2.254 (8.274) | 0.788 | -2.358 (8.598) | 0.834 |
| **Choroidal volume (mm³)** | | | | | | |
| **Outer T** | 0.135 (0.506) | 0.873 | 6.762 (8.462) | 0.630 | 2.525 (6.766) | 0.938 |
| **Inner T** | 0.257 (0.622) | 0.873 | 7.577 (9.364) | 0.630 | 3.665 (8.352) | 0.938 |
| **Central Macular** | 0.198 (0.645) | 0.873 | 5.409 (8.479) | 0.630 | 4.228 (7.659) | 0.938 |
| **Outer N** | 0.079 (0.488) | 0.873 | 13.860 (9.174) | 0.630 | 1.158 (7.904) | 0.983 |
| **Inner N** | 0.541 (0.467) | 0.873 | 5.405 (6.936) | 0.630 | -0.581 (5.193) | 0.992 |
| **Outer S** | 0.422 (0.707) | 0.873 | -8.841 (6.605) | 0.630 | 5.271 (8.038) | 0.938 |
| **Inner S** | 1.445 (0.562) | 0.174 | 6.142 (10.560) | 0.630 | -2.190 (6.802) | 0.938 |
| **Outer I** | 0.926 (0.587) | 0.650 | 6.554 (8.686) | 0.630 | 0.067 (7.011) | 0.992 |
| **Inner I** | 0.156 (0.354) | 0.873 | 8.144 (7.669) | 0.630 | 6.289 (5.859) | 0.938 |
| **Total** | -0.113 (0.397) | 0.873 | -0.286 (10.220) | 0.978 | -8.898 (10.944) | 0.938 |
| **Other choroidal parameters** | | | | | | |
| **mTCA (μm²)** | 0.072 (0.272) | 0.886 | -0.510 (5.363) | 0.998 | 1.390 (4.473) | 0.908 |
| **mLA (μm²)** | -0.036 (0.249) | 0.886 | -1.985 (5.138) | 0.998 | 0.888 (4.296) | 0.908 |
| **mSA (μm²)** | 0.034 (0.204) | 0.886 | 0.015 (5.154) | 0.998 | 2.543 (4.085) | 0.908 |
| **mCVI (%)** | -0.049 (0.054) | 0.886 | -1.038 (0.630) | 0.471 | 0.075 (0.638) | 0.908 |

Absolute interocular asymmetry was calculated as the absolute percent difference between left and right eyes normalized to their mean, with higher values indicating greater asymmetry between eyes:

$$\text{Absolute interocular asymmetry }=\frac{\text{|Left - Right|}}{(\text{Left + Right})/2}\times100$$

Each row indicates one multivariable linear regression model with absolute interocular asymmetry variable as response variable (1^st^ column). Predictor variables included into each model were: mean arterial pressure (MAP), caffeine consumption and sildenafil use. Regression coefficients (β) with HC3 heteroskedasticity-robust standard errors (SE) are presented. For continuous MAP, β represents the change in absolute asymmetry, in percentage points, per 1-mmHg increase in MAP. For caffeine and sildenafil, positive and negative β values indicate greater and lower asymmetry, respectively, compared with the corresponding unexposed reference group. Benjamini–Hochberg correction was applied separately for each group of parameters (i.e., total retinal thickness, total retinal volume, ORL thickness, RPE thickness, ONL thickness, choroidal thickness, choroidal volume, and other choroidal parameters), and separately for each predictor.

ETDRS subfields refer to the conventional Early Treatment Diabetic Retinopathy Study grid centered on the fovea: Central Macular – central subfield (within a 500-μm radius); Inner – inner ring (500–1500 μm); Outer – outer ring (1500–3000 μm). Thus “Inner T” denotes the temporal subfield of the inner ring. “Inner” and “Outer” denote topographic ETDRS rings and not retinal depth; retinal layers are designated separately as ONL, ORL and RPE.

Abbreviations: BH – Benjamini–Hochberg; CVI – choroidal vascularity index; HC3 – heteroskedasticity-consistent standard errors (type 3); I – inferior; MAP – mean arterial pressure; mCVI – macular choroidal vascularity index; mLA – macular luminal area; mSA – macular stromal area; mTCA – macular total choroidal area; N – nasal; OCT – optical coherence tomography; ONL – outer nuclear layer; ORL – outer retinal layer; PH – pulmonary hypertension; RPE – retinal pigment epithelium; SE – standard error; SFCT – subfoveal choroidal thickness; S – superior; T – temporal.
